# Personal Recollections of a Dentist of the Early Days

**Published:** 1888-01

**Authors:** L. W. Bristol

**Affiliations:** Lockport, N. Y.


### PERSONAL RECOLLECTIONS OF A DENTIST OF THE EARLY DAYS.

BY DR. L. W. BRISTOL, LOCKPORT, N. Y.

READ BEFORE THE JOINT MEETING OF THE SIXTH, SEVENTH AND EIGHTH DISTRICT DENTAL SOCIETIES AT BUFFALO, N. Y.

In the year 1850, I took charge of the office of Dr. Geo. E. Hayes, Buffalo, when he, his brother, Mr. Hayden and Dr. McBeth went across the plains to California. One morning, very early, there came to the office a richly dressed lady very much excited. She handed me a parcel, and said: "Just look at those teeth; I paid an infernal scoundrel $50 for a gold plate and they are nothing but silver, and poor silver at that." I asked her who made them. She replied, Dr. R. G. Snow. Certainly they did not look very glittering. I knew that Dr. Snow would not do a dishonest piece of work, and suspected something wrong. I stepped into the laboratory, took up a scraper, and found the plate was good gold. I asked her what she had been doing to them. She replied, nothing, except to clean them. I asked her how she cleaned them. She answered that she had put them in a tin basin with wood ashes and water, and boiled them; I asked her if the basin was clean. She said it was a bran new one, never used before, and all the gold coating had boiled off. "Now," said I, "my good woman, this is good gold plate; you have simply tinned them over. Take them back to Dr. Snow, tell him what you have done, that you came to me and I told you what was the matter, and he will take off the tinning in a short time." She brightened up and said she had always had great respect for Dr. Snow; he had always done their dentistry and she was very glad to find him still worthy of her confidence. At noon the doctor came in laughing. "That was rather a queer case you had this morning," said he; "I am very much obliged to you for the professional courtesy shown, and I shall not forget it. It is the first of the kind in all my practice."

Dr. Hayes was at that time rather exclusive. He possessed, as he thought, some valuable secrets in practice. When he went away, he drew up a power of attorney and left it in the hands of his lawyer, Tillinghast, for his wife to use in case of necessity. The doctor's first wife was a little more on the close communion order, if possible, than the doctor himself. She had seen Dr. Harvey, Dr.

Brown, and now Dr. Snow at the office. She sniffed danger to Dr. Hayes' great secrets, and came to me and said: "I observe several city dentists at the office and I do not like it. I hope you will not let them look around here. Dr. Hayes would not be pleased at that." I replied, "Mrs. Hayes, if you think Dr. Hayes' methods and secrets are in danger, you had better use your power of attorney, and I will step out. The cholera is depopulating Buffalo, business is almost suspended, the doctor's brother and Mr. Hayden have died of it at Fort Laramie, and I think the doctor himself will turn back. You talk of fleeing with your family to the Ontario hills. Now you can save all the office secrets by using your power of attorney. If you do not choose to do this, I want you to attend to your own business, and I will attend to mine; I want no more of this espionage. You send in the servant girl daily to set and watch what is said and done, and I will have no more of it. Now see your attorney and do what you think the best for Dr. Hayes' interest." She replied that she did not wish to do that, and bothered me no more.

In the year following, when I had returned to my office in Lockport, I had an experience similar to that of Dr. Snow. I had made an upper full denture on gold plate for a lady from Canada. About three months subsequently she called at my office and handed me a parcel carefully rolled up in paper, remarking: "I did not think that of you. Our family has always employed you, and thought you an honorable man and dentist, but I have found you out to be a cheat and a rascal." I unrolled the parcel and found her set of teeth looking like anything but gold. I asked her what she had been doing with them. She replied, "Nothing but scouring off the gold wash you put on." I asked her what she had been scouring them with; what kind of scouring preparation she had used. She answered that it was polishing powder that she had bought of a peddler. She was cleaning up her silverware, and thought she would give her teeth a touch, but found it took off all the "gold-coating" that had been put on, and the teeth tasted so bad that she could not wear them." I asked her if it was called "Bump's Polishing Powder." She said that was the name of it. "Well," said I, "that contains mercury and cyanide of potassium and is poison. I should have thought it would have made your mouth sore." "Oh, it did," she said, "and it is sore now." "Well," I replied, "my dear woman, you had better save your breath and anger; I am the one who should be offended. Did you not know better than to scour your good gold plate teeth with a miserable tramp's door-knob polish? Come in my laboratory and have a seat, and I will prove to your satisfaction that this is a good gold plate. Now you watch me and see that I do not put on a gold wash." I put them in the lead dish, and poured on acid and water and boiled them, then took them to the sand bath and boiled them in soda and polished them with the brush-wheel, she watching me closely. I handed her the teeth, anb she sat down and had a good crying spell over the hard things she had said of me. I gave her a good dinner and my blessing, and she departed. About two weeks after I received a letter in which she said: "I shall never hear the last from my friends and family about going clear to Lockport to have the 'Bump' taken off my teeth."

In 1849, I made a plate with five teeth on a gold base for Mrs. P. She had worn them about three months, when one night, not feeling well, she took them out and laid them on her dressing table. When she made her toilet in the morning, her teeth were missing. She ransacked the room and house thoroughly, but could not find the teeth. It was a great mystery what had become of them. She finely accused the servant girl, Jane, who had been in the family two years, of stealing them and trading them off with a peddler for old gold. The girl indignantly denied the theft and left, and got revenge by marrying a very respectable young man, and in the course of a year presenting him with a fine bouncing boy. Three years after the above occurrence, Mr. P. concluded to make some alterations in his house, and in tearing away a partition at the head of the bed, between the mop-board and the clapboards, there lay the "stolen teeth." A mouse had dragged them there. I had made Mrs. P. another set, and reflected that it was an ill wind that blew no good. Mrs. P. took the teeth and came to my office. She told me how she found them, and was feeling very badly and asked me what she should do. I told her to go and tell Jane and apologize. She said she was ashamed, but finally agreed on condition that I would go with her. I did go, told Jane that Mrs. P. had found her teeth, and how, and had now come to apologize for the wrong she had laid at her door. Well, they shook hands, and cried, first one and then the other, and then both together. I had the satisfaction of witnessing a circus without a clown, a good crying bee of two women until both were exhausted. Mrs. P. asked me if it was not awful that for three years she had accused Jane of stealing, and what I thought of the whole affair. My reply was, "God moves in a mysterious way." I left them both crying. How long the spell lasted I do not know, but I concluded that Burns was right when he said that the ways of men and women and mice "gang aft aglee."

I wish I could disabuse the public mind in regard to the practice of dentistry—that it is such an easy and lucrative profession. I have received on the average about one application a month from would-be students, whose parents or friends would say that he was not very strong, and his health was poor. I invariably answer such, "If your son or nephew has poor health, the profession of dentistry is the last business he should engage in." Only about one-third of those who enter upon dental practice ever successfully follow it for a living. From the time the dentist enters his office he has not only his own nervous system, already severely taxed, to care for, but the very worst side of his patient also, and that is not a very desirable condition for ten hours of each day. A dentist who is skilled and ambitious, and who applies himself closely to the operating chair or laboratory, lasts about five years. At the end of that time he is ready to retire and try to recover his broken health. His eye, brain, stomach, lung or liver has given out, and he must spend about all he has accumulated for medical services, or for change of air in trying to regain his health. I have been there and know how it is. For six years I worked, ate and slept under the same roof, wore a pair of office slippers, and was not a hundred yards from my office. At the end of that time I was a used-up man. I doctored with allopath, homeopath and towpath, but got no relief. One day a professor from Geddes, near Syracuse, called on me, and said: "Why, young man, you do not look as well as you used to; I will bet you are taking all kinds of medicine. What are you trying now ?" I produced from one pocket a box of Wright's Indian Vegetable pills, from another a lot of Dover's powders, from another a box of quinine, and from another a bottle of an iron mixture, and when I had emptied myself the table was about full. The Geddes philosopher gathered them all up, walked to the door and threw them into the street, saying that it was a pity to waste so much medicine, but he guessed that was the best place for it. He asked me if I had a fishpole. I said no! A gun. No! A horse. No! "Well," said he, "the first thing you do, go and get all three. Quit the office, go fishing or hunting, and into the woods, sleep on the hemlock and cedar boughs, rough it, and quit taking medicine; that is the only thing that will save your life; if you think your life worth saving, get into the open air."

Well, December came, and I joined a party of hunters and left for the old primeval woods in Canada. We arrived at a shanty in the backwoods, hired a guide and got ready for the first day's hunt. It commenced to rain, and this formed a crust on the snow. The guide said that it was a capital day for hunting, and he sent one to one place and another to another. I started out in the rainy, misty day, tramped about until two P. M., on the track of a deer, got lost, having no compass, came out on an old timber road, found a negro driving two yoke of oxen drawing a stick of timber, who was going in the direction of our shanty. We came to a clearing, when the negro saw an ox jump out and put off from the inclosure. He asked me to take his whip and keep the team in the road and he would head off the ox. I took his whip and jogged along, but I got a little too near the forward team, and the nigh ox did not like a white Yankee driver, and gave me a kick, landing me in the ditch. I got up and proceeded to teach that ox better manners. The team turned off into the gutter and upset the log. Just then the negro returned and gave me a good cursing, and I put out for the shanty. Well, here was my first day of roughing it. I had got lost, got hungry, got wet, got kicked by an ox and cursed by a negro. I expected to be dead in the morning, but got something to eat, rolled up in my Macinac blanket, went to my hemlock couch and slept the tired sleep of my first day's hunt. In the morning I was alive, had no cold, and for the first time in months had an appetite for breakfast, and did what I had not done in a year, ate a hearty meal. I spent two weeks in the wilderness, and when I returned to Lockport my friends hardly recognized me, my health and strength were so much improved.

I have but one piece of advice for you young sprouts just beginning practice. Success is what we all wish, and money we are all crazy for; but if you secure them at the price of health and life, you will make the worst bargain you ever made.

